# Does drug price-regulation affect healthcare expenditures?

**DOI:** 10.1007/s10198-016-0832-z

**Published:** 2016-09-30

**Authors:** Omer Ben-Aharon, Oren Shavit, Racheli Magnezi

**Affiliations:** Department of Management, Public Health and Health System Management Program, Bar Ilan University, 52900 Ramat Gan, Israel

**Keywords:** Drug price, Price regulation, Drug reimbursement, Healthcare expenditure, Value-based pricing, I180, H510

## Abstract

**Background:**

Increasing health costs in developed countries are a major concern for decision makers. A variety of cost containment tools are used to control this trend, including maximum price regulation and reimbursement methods for health technologies. Information regarding expenditure-related outcomes of these tools is not available.

**Objective:**

To evaluate the association between different cost-regulating mechanisms and national health expenditures in selected countries.

**Methods:**

Price-regulating and reimbursement mechanisms for prescription drugs among OECD countries were reviewed. National health expenditure indices for 2008–2012 were extracted from OECD statistical sources. Possible associations between characteristics of different systems for regulation of drug prices and reimbursement and health expenditures were examined.

**Results:**

In most countries, reimbursement mechanisms are part of publicly financed plans. Maximum price regulation is composed of reference-pricing, either of the same drug in other countries, or of therapeutic alternatives within the country, as well as value-based pricing (VBP). No association was found between price regulation or reimbursement mechanisms and healthcare costs. However, VBP may present a more effective mechanism, leading to reduced costs in the long term.

**Conclusions:**

Maximum price and reimbursement mechanism regulations were not found to be associated with cost containment of national health expenditures. VBP may have the potential to do so over the long term.

## Introduction

### Trends in drug expenditures

**Fig. 1:**
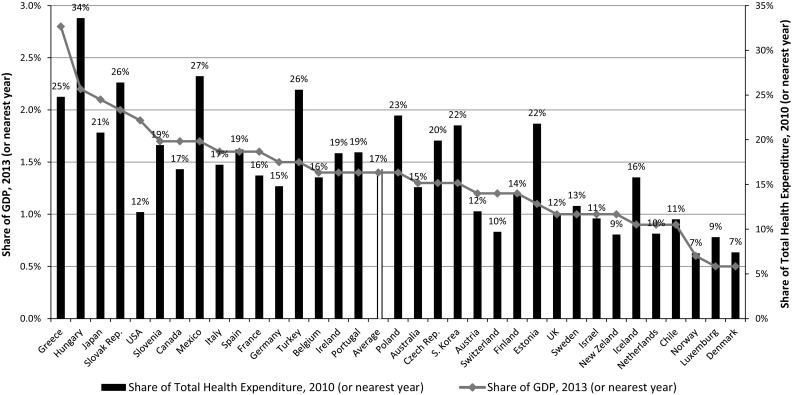
Pharmaceutical expenditures in OECD countries

Pharmaceutical spending across OECD countries was approximately US $800 billion in 2013, accounting for 17 % of total health spending [1]. Worldwide drug expenditures are projected to reach US $1.2 billion in 2017 [2]. Drug costs among OECD countries accounted for 17 % of total health expenditures in 2013, with wide variations [3]; starting at less than 10 % in Denmark and Norway, and up to more than 30 % in Hungary (Fig. 1). However, wide variations in pharmaceutical spending per capita across countries reflect differences in volume, patterns of consumption, and prices. The increasing availability of new high-cost drugs, combined with population aging, suggests that pharmaceutical expenditures may increase once again after stagnation in the past decade [1]. Several questions have been raised about accessibility, budget impact, and the legitimacy of such high prices [4]. While some high-price drugs have considerable benefits, others provide only marginal improvements to patient outcomes. Prices seem determined more by market conditions than by any concept of value in terms of clinical or additional benefits for patients.

Higher national income is generally associated with better health outcomes, although the relationship is less pronounced at the highest income levels. Studies have suggested that additional factors beyond the quality and efficiency of the health system, such as income inequality, influence outcomes [4]. The United States (US), for example, which has the highest healthcare expenditures worldwide (more than 17 % of the GDP), ranks relatively low both in life expectancy (26) and in infant mortality (31). This means that, among developed countries, factors other than financial expenses might explain better health outcomes.

As shown in Table 1, all countries reviewed in this study (except the US) supply coverage to most of their population via public health systems. Healthcare costs in the US are extremely high, while in Poland expenditures are relatively low (less than 6.5 % of GDP). Public financing of health expenditures is high in northern European countries and Japan (more than 80 %), and low (less than 65 %) in South Korea, Israel and Hungary. The US is the only country in which public financing covers less than one-half of national health expenditures. The portion of prescription medicines as a share of total health expenditures is high in Hungary (more than one-third) and low in Scandinavia (less than 10 %). In all countries, except Canada, basic insurance plans include pharmaceutical coverage for selected medicines, except in Germany and the United Kingdom (UK), where all medicines are included in the reimbursement basket, unless specifically excluded. In most countries, drugs undergo an economic evaluation prior to inclusion in the reimbursement plan; however, their impact varies from country to country, and in some cases within territories of a specific country (e.g., the Italian Health Technology Assessment body (AIFA) does not enforce its authority in southern areas). Evaluations of the prestigious British National Institute for Health and Care Excellence (NICE) are taken into account in many other countries outside the UK.

**Table 1:** Health system and prescription drug price regulation in selected countries

Country	National health system		Main health indicators (2011)			Pharmaceutical coverage in basic package		Prescription drug price regulation	Reimbursement
	National health insurance	% Coverage in public system	% Health expenditure as share of GDP	% Public expenditure as share of health expenditure	% Prescribed medicines expenditure as share of health expenditure	Yes/no	Coverage scope	Regulation type	Conducts HTAs and pharmaco-economic analysis to include drugs in reimbursement plans?
Israel	Yes	100	7.4	61	NA	Yes	Based on largest health fund basket at the time of legislation (2004) + medicines added since then	External RP	No
Australia	Yes	100	8.6	68	11	Yes	Positive medicines list covered by PBS	Internal RP	Yes
Belgium	Yes	98.8	10.6	76	11	Yes	Positive list for inpatient and outpatient drugs	Internal RP	Yes
Canada	Yes	100	10.4	70	15	No	Coverage for inpatient, most of outpatient drugs are covered via private health insurance	External RP	Yes
Denmark	Yes	100	10.5	85	5	Yes	Positive list for inpatient and outpatient drugs	None	No
France	Yes	99.9	11.1	78	13	Yes	Positive list for inpatient and outpatient drugs	External RP	Yes
Germany	Yes	88.9	10.9	77	13	Yes	Coverage to all marketed drugs unless explicitly excluded by SHI	Until 2010: none, From 2011: VBP	Yes
Hungary	Yes	100	7.8	63	34	Yes	Positive list for inpatient and outpatient drugs	Internal RP	Yes
Italy	Yes	100	8.8	79	NA	Yes	Positive list for inpatient and outpatient drugs	Internal RP	Yes (in most territories)
Japan	Yes	100	10.0	82	18	Yes	Positive list for inpatient and outpatient drugs	Internal RP	No
South Korea	Yes	100	7.1	57	17	Yes	Positive list for outpatient drugs	None	Yes (only in last decade)
The Netherlands	Yes	99.9	11.2	86	NA	Yes	Health insurance are obliged to cover drugs included in the positive list	External RP	Yes
Norway	Yes	100	8.9	85	7	Yes	Positive list for outpatient drugs	External RP	Yes
Poland	Yes	96.6	6.4	71	14	Yes	Positive list for inpatient and outpatient drugs	External RP	Yes
Portugal	Yes	100	9.7	66	NA	Yes	Positive list for inpatient and outpatient drugs	External RP	Yes
Spain	Yes	99.0	9.3	73	13	Yes	Positive list for inpatient and outpatient drugs	External RP	Yes (only in recent years)
Sweden	Yes	100	9.0	82	9	Yes	Positive list for inpatient and outpatient drugs	VBP	Yes
UK	Yes	100	8.9	NA	NA	Yes	Coverage for all marketed drugs unless explicitly excluded by NHS. Inpatient drugs are covered by hospitals according to specific separate rules	Profit control	Yes
US	No (except elderly and poor)	31.8	16.3	48	10	No	According to specific insurance plan (except for medicare and medicaid)	None	No for a reimbursement purpose (except medicaid and medicare)

* RP* Reference pricing,* VBP* value-based pricing,* HTA* health technology assessment

### Drug cost containment tools

Among developed countries, healthcare is perceived as a public product [5]. Pharmaceutical coverage is included in the basic health plans of almost all countries, except Canada, where coverage varies across territories [5]. Countries use two main complementary cost containment tools to cope with budget constraints: (1) regulation of drug prices, a mechanism intended to ensure a minimal level of drug availability at affordable prices; and (2) health technology reimbursement, a mechanism that limits accessibility in order to avoid excessive consumption.

### Regulation of drug prices

Price regulation is a mechanism for setting a maximum price on products and services in order to increase accessibility, to restrain exorbitant prices, and to slow inflation [5]. The perceived potential for manufacturers to exploit a monopoly position when facing relatively inelastic demand for drugs has led many countries to regulate prices for at least some portion of the pharmaceutical market [6]. The most common regulatory practice among OECD countries is external/internal reference pricing (RP), as shown in Table 1. External referencing (or international price benchmarking) quotes price lists from other countries that are chosen based on similarity of economic indicators. Therefore, it does not necessarily reflect the health benefit of products in the citing country. Additionally, in countries used as standard references (like the UK), the price list is not an accurate indicator of the actual price, which is set as part of confidential discount arrangements. Internal referencing (also known as therapeutic price referencing) sets a comparison to other similar drugs (based on the active ingredient and/or therapeutic indication) to examine whether the value of a new drug exceeds its marginal cost. Unlike the European Union (EU), in the US there is no tight regulation for drug prices by governance authorities [7]; nevertheless, there is de facto regulation in specific frames, such as Medicaid Health Insurance, for the low income population [6].

As of 2012, the most common price regulation method used in the countries reviewed here is external referencing (eight countries) followed by internal referencing (five countries). The US, Denmark and South Korea have no limitations on prescription drug prices. Sweden and Germany use value-based pricing (VBP). In the UK, indirect regulation is implemented via control on drug manufacturers’ profits (Table 1).

### Health technologies reimbursement

The health system structure differs among OECD countries. However, almost all (except for the US) supply basic coverage for most of the population through a national health system financed by general taxation and/or by mandatory health insurance [8]. A drug package is commonly defined by a group of selected publicly financed drugs that are dispensed for a subsidized price (co-payment). This mechanism is intended to diminish moral hazard due to excess consumption incurred when a person does not pay the full purchase price [9] while ensuring equality [10]. Germany and the UK are the only countries that automatically cover all marketed medicines unless they are specifically excluded from public financing. Most OECD countries have a structured process for evaluating new drugs or indications for existing products submitted for inclusion in public plans prior to marketing [8]. Decisions regarding reimbursement status are based on the product’s clinical efficacy, and on economic aspects such as budget impact analysis and cost effectiveness. Although Health Technology Assessment (HTA) as part of the decision process regarding reimbursement for new technologies is frequently addressed, in some cases it is a recommendation rather than mandatory [11].

### Pharmaceutical value-based pricing

In VBP, the price of a product reflects its incremental health benefit, and is considered a superior pricing approach by most scholars [12]. Porter [13] defined value as “health outcomes achieved per dollar spent”. VBP is anticipated to send manufacturers clear signals regarding developing the most efficient technologies [14]. Claxton defined VBP in the pharmaceutical sector as a price that ensures that the benefit of a new technology is greater than the cost of the current treatment [15]. Husereau and Cameron [16] suggested a wider definition of the value of new medicines to society. Early in 2002, Sweden presented a new cost-regulating mechanism characterized by cost benefit analyses for new drugs as well as generic substitutes [17]. Commencing in 2011, Germany launched the* Arzneimittelmarkt-Neuordnungsgesetz* (AMNOG) reform, in which maximum prices of reimbursed products are determined following assessment of their added medical value. Pharmaceutical companies are required to present early benefit assessment (EBA) in the negotiation process when setting the price for each new drug. The reimbursement level is determined according to its added value compared to existing therapeutic alternatives in the market [18, 19].

As far as we are aware, no previous study has examined the association between maximal price regulation or reimbursement mechanisms and national health expenditures (either as a whole, or with a specific focus on medication expenditures). In this study, we aimed to evaluate associations between different cost-regulating mechanisms and national health expenditures in selected countries. Such associations might be found not only between a specific regulating mechanism and pharmaceutical expenditure, but also between the structure of a specific health system (as reflected, for instance, in its mix of public/private financing for services), and the pricing/reimbursement tool adopted by a country.

## Methods

In order to have a significant coverage of pharmaceutical consumption, we analyzed data from nine countries that together comprise 80 % of worldwide drug expenditures [6]: US, Japan, France, Germany, UK, Italy, Canada, Spain, South Korea; as well as ten additional countries: Belgium, Hungary, The Netherlands, Portugal, Poland, Australia, Norway, Sweden, Denmark and Israel. This is a sample of all medication-consumption countries. However, they were selected to present balanced geographical coverage, including The Americas, Europe, the Far East and Oceania, which account for most of the world’s pharmaceutical expenditure. Health system characteristics and prescription drug regulation methods were reviewed, based on the World Health Organization (WHO) Health Systems in Transition series (HIT) (http://www.euro.who.int/en/about-us/partners/observatory). Health and drug expenditures indices for 2008–2012 were extracted from OECD statistical sources (http://stats.oecd.org). We examined possible associations between cost-regulating mechanisms (reimbursement and maximum price control) and national health expenditure as a whole, and pharmaceuticals specifically. Since OECD statistical sources lacked updated pharmaceutical consumption details for a relatively significant portion of the countries, in some cases the analysis was conducted until 2011 only. The following measures were evaluated: national health expenditure, public/private share of financing for prescription medicines, prescribed medicine expenditure per capita, and private expenditure for medicines as a share of private consumption. Trends over time in these expenditure indices were analyzed using two-way ANOVA with repeated measures, where years were the within-subjects variable, and regulation method was the between-subjects variable. The normality and sphericity assumptions were checked when carrying out repeated measures analysis. Due to the relatively small sample (limited by 34 countries members in the OECD), a preliminary Mauchly’s test of sphericity was conducted. In cases where the sphericity assumption was rejected, we used a more rigorous Huynh-Feldt test. In order to examine possible associations between private financing for prescription drugs and expenditures, Spearman correlations were used for the following economic indices: prescription drug expenditures as a share of GDP, as a share of national health expenditures, and as a share of private consumption.

Germany is a unique case as it changed its prescription drug regulations in 2011 when the AMNOG reform was implemented. Therefore, a separate array of tests was conducted to evaluate the impact of the regulatory change in Germany. One-way ANOVA with repeated measurements was used for 2008–2011. The trend of each economic index was tested twice: first, for all countries; and second, within all countries except Germany. Measures of prescription drug expenditures were tested as a share of GDP, as a share of total national health expenditures, and per capita.

## Results

### Associations between drug price regulation and healthcare expenditures

From 2008 through 2010, no significant change in public financing as a share of national healthcare costs for each price-regulation method was found (*F*[6, 26] = 1.635, *P* > 0.05), as well as no difference between methods (*F*[3, 14] = 0.76, *P* > 0.05). The countries with no regulation (US, South Korea and Denmark) had the lowest average financing rate and the highest internal deviation (SD 17 %). Sweden is the only country that used VBP prior to 2011 and it had the highest government financing rate.

Similar results were found regarding private expenditures as a share of total prescription drug expenditures—no change in the rate based on each regulatory method (*F*[6, 18] = 0.76, *P* > 0.05) and no difference between methods (*F*[3, 9] = 0.514, *P* > 0.05). Among countries with no regulation, average private financing was highest (35 %) and internal deviation was highest (SD 22 %).

**Fig. 2:**
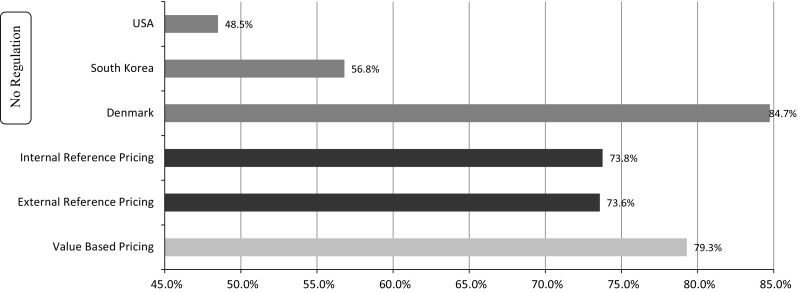
Public financing as a share of total health expenditures, 2011

As demonstrated in Fig. 2, government financing as a share of health expenditure was similar in countries with any kind of regulation (external/internal RP—74 %, VBP—79 %). In contrast, in countries with no regulation, the government portion varied widely, from very low in the US (49 %) and South Korea (57 %) to significantly high in Denmark (85 %).

The complementary picture was reflected in the examination of private financing as a share of prescription medicine expenditures: a relatively low rate was found in countries using RP (28–32 %), particularly in countries using VBP (19 %). In contrast, in countries with no regulation, the private portion was diverse: high in the US (64 %), while South Korea (30 %) and Denmark (36 %) were similar to countries with any kind of regulation.

A significant change in prescription medicine expenditure per capita for each price regulation method was found (*F*[3, 9] = 0.204, *P* < 0.05). The internal RP method had the highest increase over time, which was the source of the significant interaction.

### Associations between drug reimbursement and healthcare expenditure

No significant change in public financing as a share of national health expenditure from 2008 through 2010 (*F*[2, 26] = 1.95, *P* > 0.05) was found. Similar results were found with regard to private expenditure as a share of total prescription drug expenditure (*F*[2, 18] = 0.236, *P* > 0.05), and when testing private financing for drugs [sum of prescription and over-the-counter (OTC) drugs] as a share of private consumption.

In addition to the US, private financing for prescription drugs was high in Canada (57 %), where the public health plan does not cover drugs, and in Hungary. In all other countries, most financing for prescription medicines is public. During 2008–2010, a significant change was found in prescription medicine expenditures per capita (*F*[2, 18] = 8.49, *P* < 0.05), which increased from US $412 in 2008 to US $434 in 2009 and to US $452 in 2010. No significant correlation was found between private financing as a share of prescription drug expenditures and the following economic indices: prescription drug expenditure as a share of GDP, prescription drug expenses as a share of national health expenditure, and prescription drug expenditure as a share of private consumption.

### Impact of regulatory change in Germany on drug expenditures

**Fig. 3:**
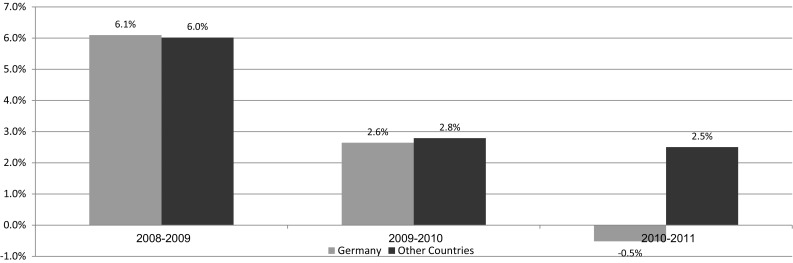
Annual change in prescription drug expenditures ($ PPP) in Germany vs. other countries

Germany is the only country that changed its regulatory policies during the study period. There was no price regulation in 2008–2010, and VBP was implemented in 2011. As shown in Fig. 3, from 2008 to 2009, and from 2009 to 2010, the change rate of prescription drug expenditures ($PPP) in Germany was similar to the equivalent rate in other countries. During 2010–2011 expenditures in Germany decreased, whereas average expenditures in other countries continued to increase.

A significant decrease in prescription drug expenditure as a share of total health expenditure from 2010 (12.31 %) through 2011 (12.07 %) was found due to Germany’s impact. No significant change in its rate as a share of GDP was found from 2010 (1.20 %) through 2011 (1.18 %); however, including Germany contributed to a decrease in this index. From 2010 through 2011, an increase in per capita prescription drug expenditures was found (4.2 %), while including Germany resulted in a more moderate increase (3.8 %).

## Discussion

The economic crisis has had a significant effect on the growth in pharmaceutical spending in many OECD countries [3]. Between 2000 and 2009, annual pharmaceutical expenditure per capita grew by 3.5 % in real terms on average in OECD countries, but, in the 2 years since 2009, the average growth became negative (−0.9 %). Annual growth rates in pharmaceutical spending were lower between 2009 and 2011 than during 2000–2009 in most OECD countries. Since the onset of the global financial and economic crisis, OECD countries have adopted a variety of drug cost containment tools. These include delisting services, administrative price cuts, erosion of pharmacy’s profits, reduction in VAT for drugs, promoting use of generic substitutes and increasing co-payments. Although this diverse tool box cannot be quantified, it provided a material contribution to the reduction in drug expenditures beyond the traditional methods for drug reimbursement and price regulation.

Countries are large, complex bodies; therefore, policy and health system changes can be implemented only slowly. This study found no significant changes over the period investigated with regard to government financing as a share of health expenditures, no change in each price regulation method and no change among methods. The last is derived from the high variability of countries with no maximum price regulation: on one side, the public financing rate for health in the US is lower than 50 %, and, on the other side, Denmark has a high 85 % rate. South Korea, with 57 %, is in between. Similar findings were noted related to the private portion as a share of total prescription drug expenditures. We found an increase over time in the drug expenditure per capita index, originating in the internal RP method. Since no significant association was found between the drug price regulation method and the level of health expenditure, it cannot be inferred that a specific control policy leads to a certain expenditure level.

As mentioned, countries with no prescription drug regulation policies are diverse. In the US, the private sector is the main supplier, and financing source for health services. This system is characterized by less efficient health outcomes and increased costs—both for health expenditures as a whole, and for drugs in particular. In contrast, Denmark has a strong public health system. Although it lacks a dedicated price regulation system, prescription drug expenditures in Denmark are low—about one-third of the OECD average, both as a share of total health expenditures (~5 %) and as a share of the GDP (0.5 %). Since population and morbidity features in Denmark are not substantially different from those of other Western European countries, the likely source for this gap is not amounts consumed (although this cannot be completely ruled out, because of lack of transparency regarding consumption in each country). Therefore, it can be logically concluded that Denmark enjoys relatively low drug prices.

Regulation of prescription drug prices is often based on citation of maximum prices in other countries. This mechanism is rather artificial, because price lists do not reflect real expense levels due to confidential commercial agreements between drug producers and health insurers. In addition, since patient co-payments are only partially connected to the drug price list, the impact of this kind of regulation on private expenditures is limited.

Sweden was a pioneer in implementing VBP early in 2002 while considering broader social aspects beyond direct drug costs. Germany launched a comprehensive reform called AMNOG in 2011, which highlights the added value of innovative drugs compared to current therapeutic alternatives. Both Sweden and Germany have modern health systems with high public funding for prescription medicines—76 % and 85 %, respectively. These findings are consistent with the known association of higher national income with better and advanced health outcomes (although the relationship is less pronounced at the highest levels of income [4]). According to the German Ministry of Health, AMNOG is a pure cost containment exercise [20]. Policymakers intended to “separate the wheat from the chaff” by differentiating between “true innovations” and products with no incremental value, as well as reconciling interests via concentrated negotiation between monopolist (producers of medicines with no alternatives) and monopsist (the body purchasing drugs for both statutory and private insurers) players. According to our results, the change of method in Germany led to lower drug expenditures for some measures (though pure causality cannot be inferred): prescription drug expenditures as a share of total health expenditures and as a share of GDP, as well as prescription drugs expenses per capita. It might be too soon to draw firm conclusions, since the reform affected prices of a limited number of products. Possibly, winds of change began in the years prior to the reform by setting prices that better reflect the drugs’ added value. In this context, it is worthwhile to bear in mind that the German legislation does not limit price-setting mechanisms to new drugs, it can also be implemented on prices of currently available drugs. The short follow-up period following the launch of AMNOG reform is a major limitation of this study. Future studies will have to validate whether the preliminary findings regarding drug expenditure in Germany were consistent over time.

The measures used in this study are common in health economics research with regard to total health expenses; yet, not for prescription medicines, e.g. many studies use a standard index of health expenditures as a share of GDP. However, we did not find any articles dealing with the portion of prescription drug expenditures as a share of GDP/total health expenditures/private consumption. In addition, the literature does not differentiate between prescription and OTC drugs, although the material difference between them is that while OTC drugs are consumer goods purchased directly by end users (and most countries do not apply interventions regarding their prices), prescription drugs are purchased via intermediation of the public health system. An OECD working paper published in 2013 [5] analyzed the perceived “value” of 14 countries when making decisions regarding reimbursement and price setting for new drugs. The paper points out the potential embedded in VBP, while in this study we went one step further, by examining the economic impact of the VBP approach as implemented in the German AMNOG act. Similarly to other studies dealing with macro-economic indicators, this paper was not able to demonstrate causality between the variables examined: reimbursement and price regulation policies on one hand and health and drug expenditure on the other. Having said that, the preliminary findings regarding Germany might hint at a potential link between these parameters.

The current analysis pertained to a relatively small number of countries. However, these countries comprise the major share of pharmaceutical consumption per capita and of pharmaceutical expenditure as a whole. In addition, several economic parameters referring to pharmaceuticals were used to overcome potential bias related to sample size.

**Fig. 4:**
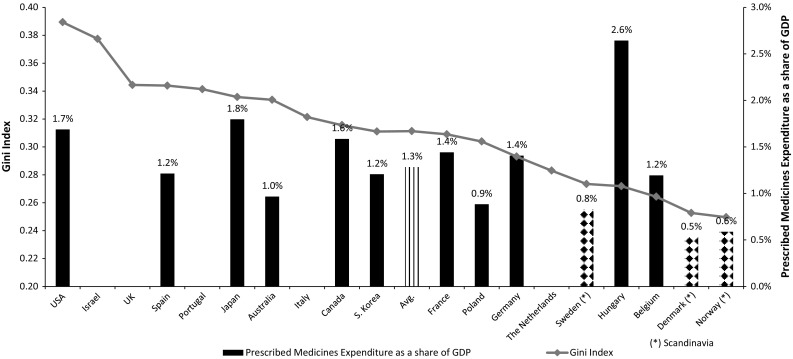
Gini Income Index and prescription drug expenditures as a share of GDP, 2011

Like Denmark, the nearby Scandinavian countries reviewed here—Sweden and Norway—are also characterized by low prescription drug expenditures. This similar outcome is achieved using completely different tools: Norway implements external RP and Sweden uses VBP, while Denmark has no maximum price regulation at all. The explanation for this phenomenon might be rooted in the low social and economic inequality in Scandinavia [21], and in a culture that does not encourage businesses to obtain extremely high profits by selling their products to government agencies. As seen in Fig. 4, these three countries are located at the lower end of the Gini Index and in prescription drug expenditures. The income inequality hypothesis is the dominant approach in discussions about health inequalities between as well as within developed nations [22]. Analysis of the relationships between income inequality and health has become a major focus of studies of the social determinants of health. The case of Scandinavia might be used as an example for potential implementation of the inequality hypothesis in the arena of pharmaceutical expenditures also.

## Conclusions

Price regulations are a natural goal for decision makers trying to meet the challenges of increasing health expenditures under budgetary constraints. However, this analysis found no correlation between various price regulation measures and health expenditures. Therefore, the results raise a question regarding the effectiveness of such policies, at least as “stand alone measures”. The results indicate that VBP might present a better option. Further research, as well as pragmatic actions, are sought in that direction.
